# Troxerutin affects the male fertility in prepubertal type 1 diabetic male rats

**DOI:** 10.22038/ijbms.2018.32678.7814

**Published:** 2019-02

**Authors:** Zohreh Zavvari Oskuye, Fariba Mirzaei Bavil, Gholam Reza Hamidian, Keyvan Mehri, Afsaneh Qadiri, Mahdi Ahmadi, Hajar Oghbaei, Amir Mansour Vatankhah, Rana Keyhanmanesh

**Affiliations:** 1 Department of Physiology, Faculty of Medicine, Tabriz University of Medical Sciences, Tabriz, Iran; 2 Drug Applied Research Center, Tabriz University of Medical Sciences, Tabriz, Iran; 3 Department of Basic Sciences, Faculty of Veterinary Medicine, University of Tabriz, Tabriz, Iran; 4 Tuberculosis and Lung Diseases Research Center, Tabriz University of Medical Sciences, Tabriz, Iran

**Keywords:** Diabetes, FSH, LH, Oxidative stress, Prepubertal, Troxerutin

## Abstract

**Objective(s):**

Diabetes can gradually cause damage to the function and structure of male gonads. This survey was conducted to investigate the effect of troxerutin

on hormonal changes, serum oxidative stress indices, and testicular function and structure in prepubertal diabetic rats.

**Materials and Methods:**

Fifty prepubertal (6 weeks old) male Wistar rats were divided into five groups including Control, Troxerutin, Diabetic, Diabetic+Troxerutin, and Diabetic+Insulin. Type I diabetes was induced by 55 mg/kg of streptozotocin intraperitoneally. The groups were treated with 150 mg/kg/day troxerutin via oral gavage or 4-6 IU/day insulin via subcutaneous injection for 4 consecutive weeks. Blood sugar (BS) and serum levels of insulin, FSH, LH, testosterone, glutathione peroxidase (GPX), superoxide dismutase (SOD), malondialdehyde (MDA), and total antioxidant capacity (TAC) were analyzed. Testis and epididymis were removed for histopathologic study and analysis of sperm parameters.

**Results:**

Troxerutin significantly reduced the BS in the diabetic group similar to insulin but could not affect insulin, FSH, or LH significantly. Troxerutin caused a significant increase in testosterone and GPX but had no significant effect on serum MDA, TAC, and SOD levels. In addition, troxerutin had a better effect than insulin on diabetes-induced testicular structural damage. Sperm analysis results also revealed that troxerutin and insulin could improve sperm number, motility, and viability in diabetic rats.

**Conclusion:**

According to these results, it can be derived that administration of troxerutin is a suitable protective strategy for side effects of diabetes in testis of prepubertal diabetic male rats.

## Introduction

Diabetes mellitus is a great endocrine and metabolic problem nowadays (1, 2). Diabetes mellitus Type 1 results from severe insulin deficiency while diabetes mellitus type 2 is characterized by insulin resistance, which may be merged with relatively reduced insulin secretion (3, 4). The report of the International Diabetes Federation in 2015 showed that the global prevalence of diabetes was estimated to be 415 million in adults and predicted that this figure will reach over 600 million in 2035 (5). 

In diabetes mellitus type 1, pancreatic beta cells are damaged by the immune system, therefore, patients must use exogenous insulin to control blood sugar and inhibit risk of developing long-term complications (6, 7). Good glycemic control can avoid its complications (8).

One of the most common complications of diabetes is sexual dysfunction (9). It has been proposed that the reproductive complications of diabetes mellitus are caused by at least two different mechanisms including endocrine disorders (10) and oxidative stress (11-13). Some of sexual dysfunctions in diabetic men are disorders in ejaculation, libido (14), erection (15), testicular tissue structure (16), sperm quality (17, 18), and testosterone and gonadotropins secretion (19, 20).

Since chemical drugs have many side effects, herbal drugs today are considered for control of diabetes complications. Herbal nutrition and major pharmaceutical companies are currently doing research on natural materials to find new herbal subjects with the least side effects (21, 22). One of these herbal subjects is troxerutin also known as vitamin P_4_. It is a tri-hydroxyethylated derivative of natural flavonoids and can be found in tea, coffee, cereal grains, and some fruits and vegetables (23). This substance can be easily absorbed by the gastrointestinal system (24) and has many biological and pharmacological activities such as anti-oxidative (25), anti-inflammatory (26), anti-fatigue (27), anti-thrombolytic (28), and anti-hyperglycemic (29) properties. Previous experiments confirmed that troxerutin has protective effects on the kidneys (23), liver (30), brain (1), and vascular injuries (24); and chronic venous insufficiency (CVI) disease could be treated by this flavonoid (31). Moreover, troxerutin could prevent nickel-induced testicular toxicity in Wistar rats (32). 

Although there are reports for the anti-hyperglycemic effects of troxerutin, we did not find any studies about the protective effects of troxerutin on the reproductive system in diabetic cases. Hence, this survey was designed to investigate the effect of troxerutin on testicular function and structure in type 1 diabetic male rats.

## Materials and Methods

***Animal and experimental design***

Fifty prepubertal (6 weeks old, weighing 90–115 g) male Wistar rats were attained from animal center of Tabriz University of Medical Sciences and transported to Drug Applied Research Center. Animals were kept in standard laboratory conditions; 12 hr light/12 hr dark cycle, 20–22 °C, 45–55% moisture, and water and food *ad libitum*. A week after transportation and adaptation, the animals were randomly divided into 5 groups (n=10); 

1. Control group (C).

2. Troxerutin group (T) which received troxerutin (Merck, Germany) 150 mg/kg/day via oral gavage for 4 weeks (24). 

3. Diabetic group (DM) (1). 

4. Diabetic group treated with troxerutin 150 mg/kg/day via oral gavage for 4 weeks (DT) (33). 

5. Diabetic group treated with neutral protamine Hagedorn (NPH) insulin 4–6 IU/day subcutaneously (DI) (34). 

For induction of diabetes, 55 mg/kg of liquefied streptozotocin (Sigma-Aldrich, Germany) in 10 mM sodium citrate (pH= 4.5) was injected intraperitoneally in DM, DI, and DT groups (35). Three days after streptozotocin injection, a blood sample of the tail vein was obtained and blood sugar (BS) was measured by means of a digital glucometer (Norditalia Elettromedicali S.r.I., Italy). If BS was more than 250 mg/dL, that animal was considered diabetic. The Ethics Committee of Tabriz University of Medical Sciences has supported all experimental procedures (No: IR.TBZMED.REC.1395.584).

***Sampling***

On the last day of experiments, animals were deeply anesthetized with intraperitoneal injection of a combination of ketamine and xylazine (80 and 12 mg/kg, respectively). Five milliliters of blood samples, taken from the inferior vena cava, were centrifuged at 3000 rpm for 10 min at room temperature. Then, serum aliquots were isolated and were kept at -80 ^°^C for later hormonal and oxidative stress analysis. Finally, animals were sacrificed by decapitation and the left testis was removed. Length (longitudinal radius), width (transversal radius), and height (perpendicular to the transversal radius) of each testis was measured by caliper and after macroscopic evaluation tissue samples were assessed after fixation in 10% buffered neutral formalin. 

**Figure 1:**
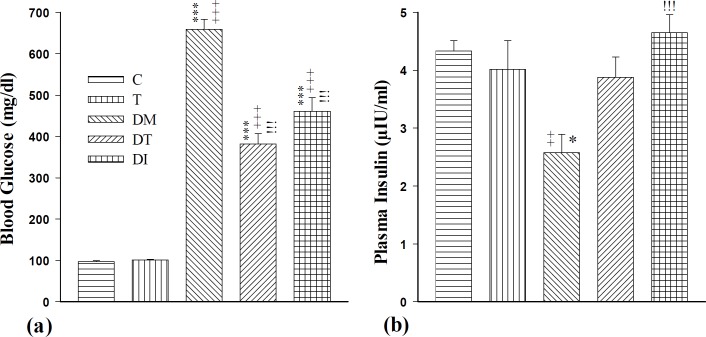
The effect of troxerutin on blood glucose (a) and plasma insulin (b) in prepubertal type 1 diabetic male rats (n=10). Data are expressed as mean±SEM. Data were analyzed by using one way ANOVA followed by Tukey’s *post hoc* test. Statistical differences between control and different groups: +++; *P<*0.001, ++; *P<*0.01, Statistical differences between troxerutin and different groups: ***; *P<*0.001, *; *P<*0.05, Statistical differences between diabetic and different groups: !!!; *P<*0.001. Control (C), troxerutin (T), diabetic (DM), diabetes+troxerutin (DT), and diabetes+insulin (DI)

**Figure 2:**
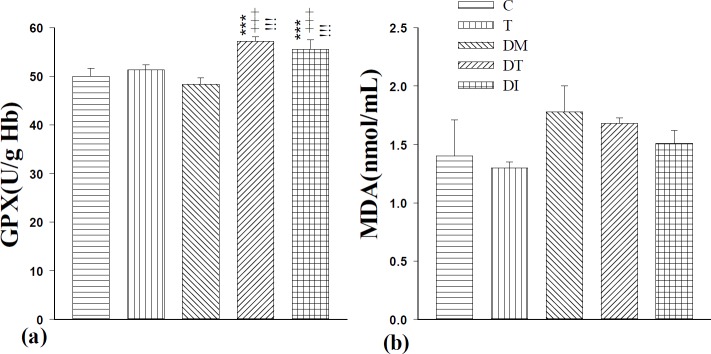
The effect of troxerutin on serum glutathione peroxidase (GPX, a) and malondialdehyde (MDA, b) in prepubertal type 1 diabetic male rats (n=10) for 4 weeks. Data are expressed as mean±SEM. Statistical differences between control and different groups: +++; *P<*0.001, Statistical differences between troxerutin and different groups: ***; *P<*0.001, Statistical differences between diabetic and different groups: !!!; *P<*0.001. Control (C), troxerutin (T), diabetic (DM), diabetes+troxerutin (DT), and diabetes+insulin (DI)

**Figure 3:**
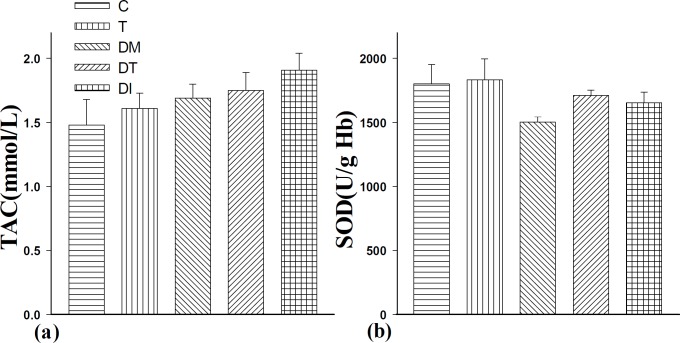
The effect of troxerutin on total antioxidant capacity (TAC, a) and superoxide dismutase (SOD, b) in prepubertal type 1 diabetic male rats (n=10) for 4 weeks. Control (C), troxerutin (T), diabetic (DM), diabetes+troxerutin (DT), and diabetes+insulin (DI)

**Figure 4:**
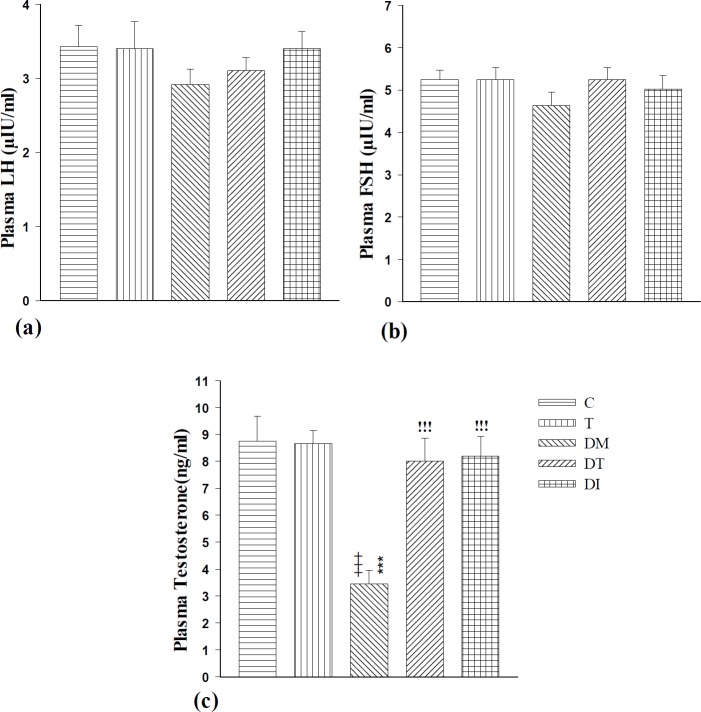
The effect of troxerutin on LH (a), FSH (b), and testosterone (c) in prepubertal type 1 diabetic male rats (n=10) for 4 weeks. Data are expressed as mean±SEM. Statistical differences between control and different groups: +++; *P<*0.001, Statistical differences between troxerutin and different groups: ***; *P<*0.001, Statistical differences between diabetic and different groups: !!!; *P<*0.001. Control (C), troxerutin (T), diabetic (DM), diabetes+troxerutin (DT), and diabetes+insulin (DI)

**Figure 5:**
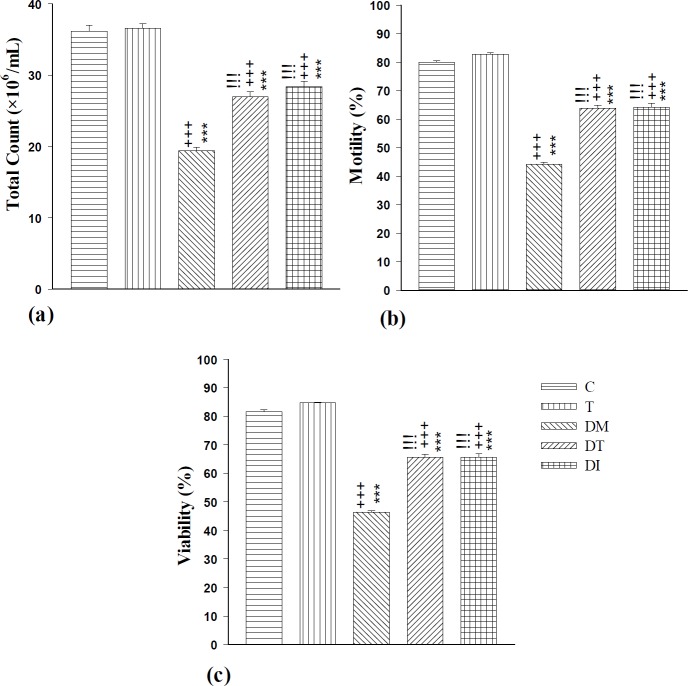
The effect of troxerutin on total count (a), motility (b), and viability (c) of sperms in prepubertal type 1 diabetic male rats (n=10) for 4 weeks. Data are expressed as mean±SEM. Statistical differences between control and different groups: +++; *P<*0.001, Statistical differences between troxerutin and different groups: ***; *P<*0.001, Statistical differences between diabetic and different groups: !!!; *P<*0.001. Control (C), troxerutin (T), diabetic (DM), diabetes+troxerutin (DT), and diabetes+insulin (DI)

**Figure 6:**
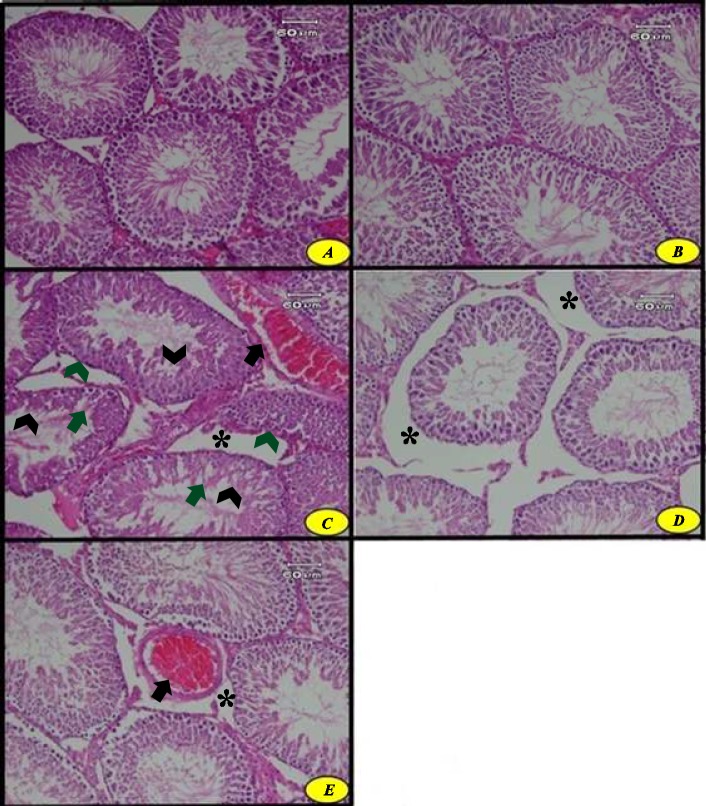
The effect of troxerutin on histological structure of testis in different groups (H&E, ×200) in prepubertal type 1 diabetic male rats (n=10) for 4 weeks. Control (A), troxerutin (B), diabetic (C), diabetic and troxerutin (D), and diabetic and insulin (E). An increase in interstitial space (*), vascular congestion (black arrow), presence of many vacuoles in the germinal epithelium (green arrow), destruction of germinal epithelium of seminiferous tubule (black arrowhead), shrinkage of tubule wall (green arrowhead) are observed

**Figure 7:**
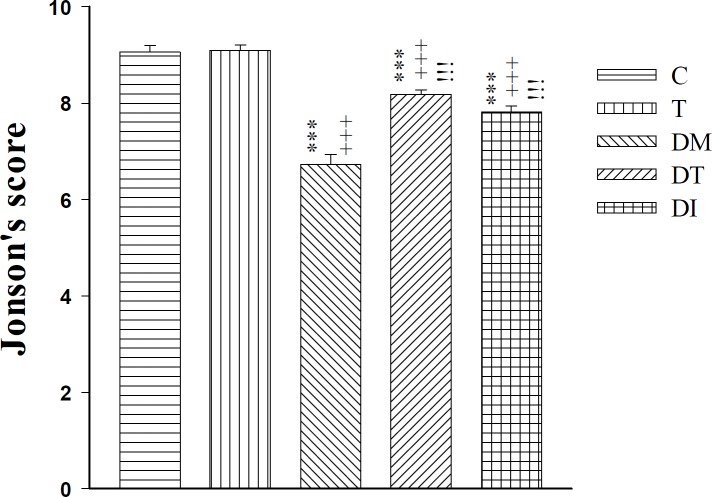
The effect of troxerutin on Johnsen’s score for histopathological evaluation of testis tissues in prepubertal type 1 diabetic male rats (n=5) for 4 weeks. Data are expressed as mean±SEM. Statistical differences between control and different groups: +++; *P<*0.001, Statistical differences between troxerutin and different groups: ***; *P<*0.001, Statistical differences between diabetic and different groups: !!!; *P<*0.001. Control (C), troxerutin (T), diabetic (DM), diabetes+troxerutin (DT), and diabetes+insulin (DI)

**Table 1:** The effect of troxerutin on sperm motility grade in prepubertal type 1 diabetic male rats (n=10) for 4 weeks.

Motility Grades	Scores in groups				
	% (Mean±SEM)				
	Control	T	DM	DT	DI
Grade A (fast progressive)	48.6±0.68	49.6±0.24	20.4±0.93 +++ ***	30.6±0.87 +++ *** !!!	29.4±0.60 +++ *** !!!
Grade B (slow progressive)	22.0±0.55	25.0±0.63	12.2±0.58 +++ ***	28.0±0.89 +++ *** !!!	25.6±1.07 +++ *** !!!
Grade C (non-progressive)	8.6±0.24	8.2±0.37	5.8±0.37 ++ **	5.4±0.24 ++ **	8.2±0.20 !! ##
Grade D (immotile)	20.8±0.86	17.2±0.58	61.6±1.32 +++ ***	36.0±1.00 +++ *** !!!	36.8±1.24 +++ *** !!

Data are expressed as mean±SEM. Statistical differences between control and different groups:

+++ ; *P*<0.001,

++ ; *P*<0.01, Statistical differences between troxerutin and different groups:

*** ; *P*<0.001,

** ; *P*<0.01, Statistical differences between diabetic and different groups:

!!! ; *P*<0.001,

!! ; *P*<0.01, Statistical differences between DT and DI groups:

## ; *P*<0.01. Control (C), troxerutin (T), diabetic (DM), diabetes+troxerutin (DT), and diabetes+insulin (DI).

***Hormonal analysis of serum***

The serum levels of insulin (Shanghai crystal Day Biotech Co, LTD, China, for insulin with 0.05 ng/ml sensitivity), FSH (Bioassay Technology Laboratory, China, with 0.12 mIu/ml sensitivity), LH (Bioassay Technology Laboratory, China, with 0.051 mIu/ml sensitivity) and testosterone (Diametra Co, Italy, with 0.07 ng/ml sensitivity) were measured using enzyme-linked immuno-absorbent assay according to the manufacturer’s instructions (8). 

***Oxidative stress measurement***

Serum level of glutathione peroxidase (GPX), superoxide dismutase (SOD), malondialdehyde (MDA), and total antioxidant capacity (TAC) were analyzed. The activity of GPX was measured using Randox kit, United Kingdom. SOD was evaluated using a spectrophotometric method on the basis of the inhibition of a superoxide-induced reduced nicotinamide adenine dinucleotide (NADH) oxidation and MDA was measured using the thiobarbituric acid (TBARS) and colorimetric method (33). TAC was measured using the Randox Kit according to the protocols of the manufacturer. 

***Sperm parameters***

Immediately after the last intervention, animals were anesthetized and the testes, epididymis and vas deferens were removed for determination of the spermatozoid parameters. To calculate the epididymal sperm storage, epididymis were isolated from the testes. Accordingly, epididymis was cut from the tail, and the sperm content of epididymis was aspirated into a pre-weighed pipette. Then the pipette was re-weighed to measure the weight of the aspirated fluid (36). 

A 1 ml sample of the suspension containing sperm extracted from the epididymis tail was diluted with 20 ml of Ham’s F10 solution and the sperm count and motility were determined at ×400 magnification. Subsequently, the total mean of motile sperms in ten fields was expressed as a percentage of motility according to the World Health Organization (WHO) guidelines (37).

Determination of the spermatozoa viability percentage was also performed according to the WHO guidelines, using eosin (1%) and nigrosin (10%) staining. Briefly, 1 ml sperm suspension was mixed with 2 ml of eosin. After incubation at 37 ^°^C for 30 sec, an equal volume of nigrosin was added to this suspension. Then the percentage of viable sperms was calculated in different groups under a light microscope (3).

Moreover, the total number of sperms was counted in a hemocytometer. Concisely, the number of sperms in diluted sperm solution was counted in five large squares of a hemocytometer. Then the sperm count per milliliter was calculated (38, 39).

For evaluation of sperm motility, at least 200 spermatozoa in 100 μl sperm suspension were evaluated under a light microscope and scored from A to D according to the WHO manual including (40, 41): grade A as fast progressive; grade B as slow progressive; grade C as non-progressive, and grade D as immotile sperm.

***Histological analysis***

Fixed testicular tissue samples were dehydrated in an ascending graded series of ethylic alcohol, cleared in xylol, and impregnated in paraffin. The testis was cut by a rotary microtome to 5 μm thin sections and was stained by hematoxylin and eosin (H&E) according to a previously described protocol. At least 10 sections were checked for each animal with a light microscope. Testis and seminiferous tubules were evaluated histologically (3).

Germinal epithelium organization and quality of 100 seminiferous tubules per animal were analyzed by Johnsen’s score from 1 to 10 according to the following criteria (41):

Score 10: full spermatogenesis with regular germinal epithelium; Score 9: disorganized germinal epithelium with many spermatozoa; Score 8: existence of only a little spermatozoa; Score 7: no spermatozoa but many spermatids existed; Score 6: no spermatozoa and only a few spermatids were observed; Score 5: no spermatozoa, no spermatid but many spermatocytes existed; Score 4: only a few spermatocytes were observed; Score 3: only spermatogonia were present; Score 2: Sertoli cells were observed but no germ cells were present; Score 1: seminiferous tubule without any cells. All evaluations were done by an expert technician who was blinded to our study.

***Statistical analysis***

The results were expressed as mean ± SEM (standard error of the mean). Statistical analysis was performed by SPSS software version 22 (IBM company, SPSS Inc., 2010). One-way analysis of variance (ANOVA) followed by *post hoc* Tukey’s test was used to assess the statistical significance of data between different groups. It was considered significant if *P*<0.05.

## Results

***The levels of blood glucose and serum insulin***

The blood glucose levels in DM, DT, and DI groups increased significantly compared to the control and troxerutin groups (*P*<0.001). Both insulin and troxerutin treatment significantly diminished the blood glucose levels in comparison with the diabetic group (*P*<0.001) (Figure 1a).

Injection of streptozotocin in the DM group reduced insulin level compared to C and T groups (*P*<0.01 to *P*<0.05). There was an increased insulin level in the DI group compared to diabetic rats (*P*<0.001), however, administration of troxerutin nonsignificantly increased the insulin level compared to the DM group (Figure 1b).

***The levels of serum glutathione peroxidase (GPX) and malondialdehyde (MDA)***

Induction of diabetes did not show any effect on the GPX level; however, in DI and DT groups, administration of insulin and troxerutin increased GPX level significantly (*P*<0.001). It must be mentioned that insulin and troxerutin did not have a significant difference in this regard (Figure 2a).

Although induction of diabetes increased the MDA level in comparison to the controls, this increment was not statistically significant. Treatment with insulin and troxerutin in DI and DT groups nonsignificantly decreased the level of MDA. There was no significant difference between DI and DT groups (Figure 2b).

***The serum levels of total antioxidant capacity (TAC) and superoxide dismutase (SOD)***

Induction of diabetes and administration of troxerutin and insulin did not change the TAC level significantly compared to the C group (Figure 3a).

Although induction of diabetes decreased the SOD level in comparison to the controls, it was not statistically significant. Treatment with insulin and troxerutin in DI and DT groups nonsignificantly increased the level of SOD in comparison to the DM group. There was no significant difference between treated diabetic groups (Figure 3b).

***The serum levels of LH, FSH, and testosterone***

The serum testosterone level decreased significantly after diabetes induction compared to C and T groups (*P*<0.001), whereas LH and FSH serum levels decreased nonsignificantly. Treatment with insulin and troxerutin in DI and DT groups increased testosterone level significantly in comparison to diabetic rats (*P*<0.001), although the increments in the serum levels of LH and FSH in these groups were not statistically significant. There were not any significant differences between DT and DI groups (Figures 4a, b, and c). 

***Sperm parameters***

The induction of diabetes reduced the total number, motility, and viability of sperms in comparison to C and T groups (*P*<0.001). Troxerutin could not affect these parameters in control rats. Administration of troxerutin and insulin significantly improved all of these parameters compared to the DM group (*P*<0.001). There were no significant differences between DT and DI groups (Figures 5a, b, and c). 

***Sperm motility grade***

Induction of diabetes significantly decreased the percentages of fast progressive, slow progressive and non-progressive sperms (*P*<0.001 to *P*<0.01) but increased the percentage of immotile sperms (*P*<0.001) compared to control and troxerutin groups. Treatment with troxerutin significantly increased the percentages of fast progressive and slow progressive sperms but decreased the percentage of immotile sperms (*P*<0.001). Moreover, in the DI group, insulin administration increased the percentages of fast progressive, slow progressive, and non-progressive sperms (*P*<0.01 to *P*<0.001) and decreased the percentage of immotile sperm (*P*<0.001) compared to the DM group. The percentage of non-progressive sperms of DT and DI groups was significantly different (*P*<0.01, Table 1).

***Histological analysis of testis***

Histological studies of testis tissue demonstrated that the structure of the testis and seminiferous tubule are completely normal in the control and troxerutin-treated groups (Figures 6A and B). Microscopical analysis revealed that diabetes induction resulted in structural disturbance of the animals’ testis including increased interstitial space and vascular congestion and destruction of germinal epithelium of seminiferous tubule. Existence of many vacuoles in germinal epithelium caused rupture in the cells integrity of this tissue. Irregular shape and shrinkage of seminiferous tubules basement membrane and disturbance of cellular arrangement and organization could be observed in diabetic rats (Figure 6C).

Histological studies of the testis in the diabetic group treated by troxerutin showed that the tissue structure was improved relatively compared to the diabetic group. The germinal epithelium of seminiferous tubule had proper structure and was similar to the control group. Increased interstitial space could be still observed in this group but vascular congestion was completely resolved (Figure 6D).

Microscopic studies also revealed that treatment by insulin in the diabetic group resulted in evident structural improvement but increased volume in interstitial space could be still observed. Vascular congestion was less than in the diabetic rats but it was not totally eliminated. The effect of insulin administration on the improvement of structural changes of testis tissue was less compared with the group treated by troxerutin for 4 weeks (Figure 6E).

Johnsen’s score analysis illustrated a significant decrease in all diabetic rats (D, DT, and DI groups) compared to C and T groups (*P*<0.001). Treatment with troxerutin and insulin in DT and DI groups induced a significant increment compared to the DM group (*P*<0.001) (Figure 7).

## Discussion

Our results indicated that treatment of diabetic animals by insulin and troxerutin resulted in significant increase of GPX, testosterone, total number, viability, and motility of sperm and Jonson’s score, although it reduced the blood sugar. The previous studies have shown that high blood sugar levels can affect fertility (42, 43). The results of a study have proven that hyperglycemia increased the testicular inflammatory cytokines (44). Other studies also suggested that hyperglycemia caused excessive production of ROS (45, 46) followed by the destruction of the testicular membrane (47).

Nowadays various drugs are used to treat diabetes, which may have a negative effect on other organs in addition to lowering blood glucose levels. One study reported that the use of sulfonylureas has led to apoptosis in beta cells and the failure of long-term treatment (48). Researchers also found that metformin and glibenclamide caused a significant reduction in the number and motility of the sperm and testicular damage, due to elevated lipid peroxidation and decreased antioxidant status in the testes (49). 

Because of these side effects, scientists have tried to use alternative herbal drugs for controlling complications of diabetes. One of these medicines is troxerutin. Regarding the positive effects of insulin on fertility, we decided to compare the drug with insulin. Insulin stimulates various functions of Sertoli cells, such as transferrin secretion, DNA and protein synthesis, glycine metabolism, lactate production (50, 51), and differentiation of spermatogonia through insulin-like growth factor receptor (IGF-1)(52).

In a study in 2014, increased sensitivity to insulin in the presence of troxerutin has been reported as a major finding (53). Another study has also shown that routine supplementation effectively relieves symptoms of metabolic syndrome. In addition, they showed that the administration of troxerutin in high-cholesterol-fed type 2 diabetic rats reduced the blood glucose levels, consistent with our results (54).

Kawamura *et al.* reported that SOD glycosylation percentage was significantly elevated in diabetic people compared with controls. The activity of glycosylated SOD is less than natural SOD (55). A study showed that MDA level of seminal plasma in diabetic men with normal sperm is more than that of non-diabetic men. Also, it has been shown that diabetic men have lower levels of TAC compared to non-diabetic men (56). However, the current study showed that administration of troxerutin (150 mg/kg) in immature diabetic rats had no significant effect on SOD, MDA, and TAC levels of serum in comparison to the diabetic group, but led to increment in serum level of GPX in comparison to the diabetic group. Previous study has also revealed that administration of troxerutin to diabetic rats will not have a significant impact on the increase of SOD in comparison to those which had not received troxerutin, although the serum level of GPX significantly increased (24), which is consonant with the results of our study. However, the current results did not coincide with the findings of Fan *et al* who investigated the effect of troxerutin on D-galactose-induced renal injury in mice. These results indicated that it could increase the activity of antioxidant enzymes and reduce the lipid peroxidation products (23). The reasons for such differences can be attributed to the duration and severity of diabetes, method, and dosage of drug administration and method of diabetes induction. 

Ballester *et al.* observed that induction of type 1 diabetes by streptozotocin for 3 months disturbed the function of Leydig cells and decreased the serum level of testosterone. This could be due to lack of stimulation effect of insulin on these cells. They also showed that the serum level of FSH and LH would also decrease in such conditions (57). In our study, the level of testosterone decreased significantly, although the levels of LH and FSH were decreased nonsignificantly. It seems that the duration of the experimental period of our study (4 weeks) can be the reason for these results, as the work of Ballester *et al.* (57) was conducted for 3 months. On the other hand, the age of rats could also make a difference. They worked on adult rats whereas our study was performed on prepubertal rats. 

Previous study has revealed that diabetes can cause severe abnormalities in sperm by increasing the oxidative stress in testis and epididymis tissues (58). It has been shown that sperm cells of mammalians contain high levels of lipids with high unsaturated fatty acids. On the other hand, spermatozoa use lipids as the main material for the peroxidation process. This can make the testis, epididymis, and released sperms in the seminiferous tubule a suitable site for production of free radicals as the result of lipid peroxidation during diabetes. The high rate of cell proliferation in germinal epithelium of the seminiferous tubule and reduction of anti-oxidative defense during diabetes can even intensify this issue (59). It has been also shown that hyperglycemia can increase the production of free radicals by increased glycolysis, activation of the sorbitol pathway in the cell, glucose self-oxidation, and proteins non-enzymatic glycation (45, 46), which is compatible with our findings in this investigation.

The results of qualitative and quantitative analysis of sperms in our study revealed that induction of diabetes by streptozotocin not only can affect the viability and the total number of sperms but also it can reduce the quality of sperm motility. Previous studies also suggested that diabetes and its consequent hyperglycemia can cause a reduction in quality and quantity parameters of sperm (3), disturb the spermatogenesis process (60), and also decrease the sperm motility (57) by decreasing the production rate of testosterone.

Troxerutin has numerous biological protective effects against oxidative, fibrinolytic, inflammatory, γ-radiation, and diabetic damage and cancer (28, 54). It acts by affecting reactive oxygen species (ROS) and enzyme activities, probably indirectly by affecting the antioxidants acting on enzyme activities (61). The present study showed that troxerutin administration, similar to insulin, could relatively improve the diabetes-induced decrease in quantity and quality parameters of sperms. It seems that troxerutin can decrease the oxidative stress and blood sugar and relatively inhibit the side effects of diabetes on quality and quantity indices of sperm. 

The results of this survey showed that induction of diabetes caused severe damage to the testicular tissue structure. Microscopical study showed that diabetes induced vascular hyperemia in the interstitial tissue of testis and increased the interstitial space of the seminiferous tubules due to interstitial edema. Kolahian *et. al.* also reported these events in their study (3). The outcomes of our study displayed that induction of diabetes caused severe degeneration of spermatogenic cells as a result of structural changes in the germinal epithelium of seminiferous tubules. The disappearance of spermatogenic cells leads to the observation of many vacuoles in the germinal epithelium. In another study these reports were also observed (62). The results of the sperm analysis indicated that diabetes reduced testicular function and normal sperm production. The results of present investigation also showed that insulin or troxerutin therapy can importantly inhibit structural changes of the testis, surprisingly the effect of troxerutin was better than insulin and decreased the vascular congestion in testis, which was also consonant with the results of sperm analysis. So possible mechanisms that may be proposed for these effects of troxerutin include anti-inflammatory drug effect (28, 54), reduction of blood glucose (54, 63), improved insulin sensitivity (53), and indirect effect on antioxidants (61) because oxidative stress disrupts fertility (47). 

## Conclusion

According to these results, it can be concluded that administration of troxerutin is a suitable protective strategy for side effects of diabetes in testis of prepubertal diabetic male rats.
